# The examination of biophysical parameters of the skin in Polish Konik horses

**DOI:** 10.1371/journal.pone.0250329

**Published:** 2021-06-21

**Authors:** Agnieszka Cekiera, Jarosław Popiel, Marta Siemieniuch, Zbigniew Jaworski, Malwina Slowikowska, Natalia Siwinska, Agnieszka Zak, Artur Niedzwiedz

**Affiliations:** 1 Department of Internal Medicine and Clinic of Diseases of Horses, Dogs and Cats, Faculty of Veterinary Medicine, Wrocław University of Environmental and Life Sciences, Wrocław, Poland; 2 Department of Reproductive Immunology and Pathology, Institute of Animal Reproduction and Food Research of Polish Academy of Sciences, Olsztyn, Poland; 3 Research Station of Institute of Animal Reproduction and Food Research of PAS, Ruciane-Nida, Poland; 4 Department of Horse Breeding and Riding, University of Warmia and Mazury, Olsztyn, Poland; University of Lincoln, UNITED KINGDOM

## Abstract

This study aimed to assess the biophysical parameters of the skin in Polish Konik horses (Polish primitive horses). According to the authors, this is the first assessment performed on such a wide scale in this group of animals. The evaluation carried out is innovative both with regards to the breed of the animals and the wide scope of the physicochemical skin assessment. The study group comprised mares, stallions and geldings, and the evaluations concerned transepidermal water loss, corneometry, pH, skin temperature assessment and mexametry. These parameters were assessed in five skin regions: the lips, the right ear, the prosternum, the right side of the neck and the chest. The measurements were taken after spreading the hair apart, with the use of a Multiprobe Adapter System (MPA^®^) and dedicated probes (Courage + Khazaka electronic GmbH, Cologne, Germany). The measurements revealed statistically significant differences in the values of transepidermal water loss in the lips in mares compared with stallions (P = 0.023) and also in stallions compared with geldings (P = 0.009). Corneometry showed significantly higher results in the neck region in mares compared with stallions (P = 0.037) and the prosternum areas in mares and geldings compared with stallions (P = 0.037 and P = 0.018). Skin pH measurement on the right side of the neck rendered significantly higher values in stallions than in mares (P = 0.037). In geldings, the skin temperature was significantly higher than in stallions (P = 0.049). Once the appropriate physicochemical values for specific animal species and breeds are determined, non-invasive methods of skin examination in many diseases and also methods of evaluation of the efficacy and/or adverse effects of applied medications can be established.

## Introduction

In veterinary dermatology, there is a constant need to develop new, primarily non-invasive methods of skin assessment. There is a demand for new techniques which can not only assess the skin condition of an animal but also evaluate treatment progress in skin diseases or the efficiency of new generation drugs. Biophysical skin assessment, although widely applied in human medicine and cosmetology, is still rarely used in veterinary medicine. So far, the use of the following properties has been studied in animals: transepidermal water loss (TEWL), skin hydration (SH), pH level and intensity of skin erythema, yet the majority of assessments have been made in dogs and cats [1–5]. Assessments of skin properties in horses have only recently become the subject of scientific interest for researchers–they have studied TEWL, SH, pH and the probable influence of animal breed and hair removal on the results obtained. Parameters can be used to identify specific skin problems using non-invasive techniques. These can be used to decide which therapeutic method to use and for evaluation of effectiveness of local and general treatment in atopic dermatitis, pyoderma and other skin diseases. These methods can be also used as a marker of skin integrity and damage, trauma, metabolic disorders and seborrhoea. Identification of very specific skin abnormalities can be a very useful tool to improve treatment methods and treatment outcomes. In a study comprising four horse breeds (‘Felin’ ponies, Polish Konik horses, Polish draft horses and Wielkopolska horses) differences in TEWL values have been shown, yet only in some limited regions of the body–the most significant differences were observed in the lumbar and inguinal regions [6]. Specifically, TEWL can evaluate epidermal damage, especially important in atopic dermatitis. Corneometry parameters will change in atopic dermatitis, trauma and metabolic disorders. Skin pH is proven to change in atopic dermatitis, seborrhoea and pyoderma. There are still a lot of questions regarding horses because of limited data and further studies must be conducted–first of all in healthy horses to determine the basic values that will make it clear in which cases the values are changed. The authors also wanted to compare the biophysical parameters between gender as there are some studies in human medicine revealing that skin pH, for instance, can be different in females and males. As a comparison of parameters has not been conducted in horses, it seems important to do so as the results could be under the influence of hormones etc.

This study aimed to define reference values for biophysical parameters in the Polish Konik horse. An additional objective was to evaluate the impact of the gender of an animal on the biophysical parameters of the skin. In this study, we explored the hypothesis that skin biophysical parameters may differ depending on the gender of an animal within the same horse breed.

Studies carried out in humans provide contradictory results. On the one hand, it is known that there are differences in the values of such parameters, depending on gender and ethnic origin, but on the other hand, it is known that these results should be compared within groups of individuals with a similar environment and lifestyle [7, 8]. For this reason, the authors of the present study concentrated on horses of a specific breed abiding in identical environmental conditions. The results of studies carried out by other authors indicate only to slight differences within animal groups and the examined body regions [6, 9, 10].

TEWL is indicative of the integrity of the skin barrier. The assessment of TEWL is used to evaluate skin damage, including damage to the superficial layers of the epidermis [11, 12]. It has been shown that TEWL values increase in some skin diseases, such as atopic dermatitis in humans and in dogs [13]. Additionally, the role of ceramides in the maintenance of the skin barrier has been observed and thus, a deficit of ceramides contributes to an increase in TEWL, but there is a need for further research in horses [12].

## Materials and methods

### Ethical approval

All examinations performed in this study were non-invasive and are routinely performed in everyday medical practice. Following existing law applicable in Poland, based on the Experiments on Animals Act from 15 January 2015 (Journal of Laws of the Republic of Poland, 2015, item. 266), non-invasive clinical studies do not require ethical approval.

### Animals

The study group comprised 21 adult Polish Konik horses, divided into three groups with regards to gender: seven females (mean age: 4.4 years; range 3–7), 14 males, including seven geldings (mean age: 8.7 years; range 3–13) and seven stallions (mean age: 6.7 years; range 2–13). To date there are no data indicating that age can or cannot affect skin parameter values, so the study was comprised only of a group of adult horses. All the animals were kept at the Research Station of the Institute of Animal Reproduction and Food Research of the Polish Academy of Sciences. The horses were maintained in a stable–pasture system with full-time access to hay, water and mineral licks, and were fed oats in a dosage based on their nutrient requirements, body weight and level of physical activity. To date there are no data suggesting that body weight or condition score can or cannot influence skin parameter values, so the study comprised a group of healthy animals. The study was conducted three hours after feeding and water intake. Exclusion criteria included systemic diseases or skin lesions, and, in the case of mares–pregnancy, gestation or lactation periods. In all cases, no disease symptoms were observed within the previous 30 days, and also no treatment, either systemic or local, had been applied. All animals underwent a physical examination and a detailed dermatological assessment. In the dermatological evaluation, special attention was paid to the presence of alopecia and skin eruptions. Moreover, one condition for inclusion into the study group was a negative result of a mycological culture of hair samples from the regions selected for physicochemical assessment–the samples were cultured with the standard method, on Sabouraud agar, in a quality-assured veterinary laboratory. Before the physicochemical skin assessment, the horses remained in the stables for a minimum period of 90 minutes for acclimatisation. The climatic conditions in the premises were constant–temperature: 21.3°C and humidity: 58%.

### The assessment of biophysical skin parameters

**Fig 1:**
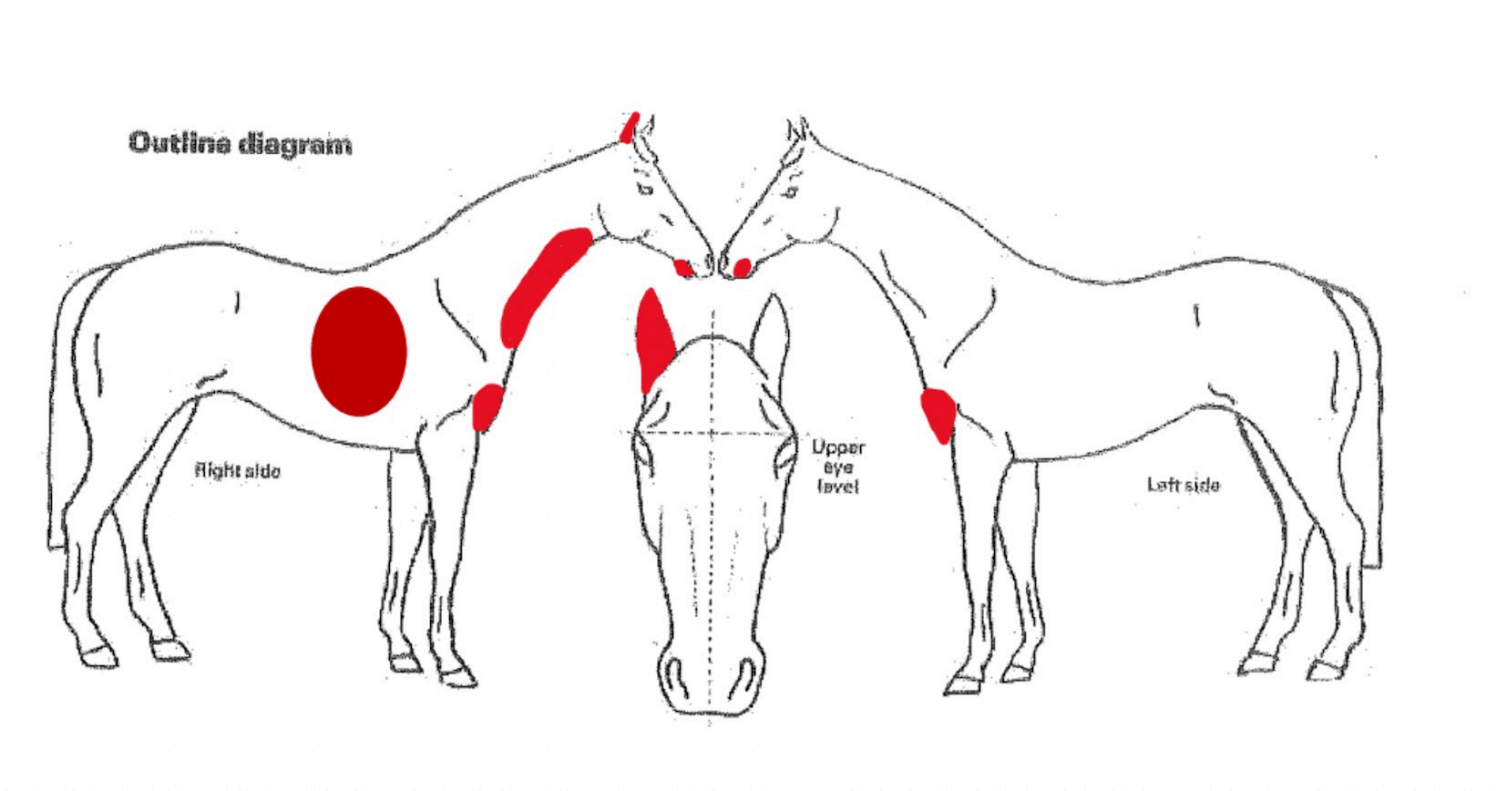
Assessed skin regions: The right side of the neck, the right side of the chest, the lip area, the right auricle and the prosternum.

The measurement of biophysical skin parameters was made using a Multiprobe Adapter System (MPA^®^) and dedicated probes (Courage + Khazaka electronic GmbH, Cologne, Germany). The regions were chosen based on the fact that previous studies were made in these body areas. Each parameter was assessed in each horse in five skin regions: the right side of the neck, the right side of the chest, the lip area, the right auricle and the prosternum (Fig 1). The skin was not washed and the hair was not clipped before assessment, but when the skin was strongly soiled, the hair was gently combed. For the measurements, the hairs were spread apart. The hairs were not clipped, as in most conducted studies there was no information regarding how clipping can affect the skin parameter values. Hairs are the natural barrier of the skin and have a role in maintaining humidity. The authors wanted to investigate normal healthy skin and hair removal could be a disturbing factor. The only proof that clipping improves the measuring technique is in case of TEWL. The authors decided that, in this case, hair clipping can influence the other results.

The TEWL measurement was made with a Tewameter®TM 300 probe with a continuous measure for 30 seconds. Corneometry was performed with a Corneometer^®^ CM 825 probe. The skin pH was measured with a Skin-pH-Meter PH 90 probe and body temperature with a Skin-Thermometer ST 500 probe. The mexametry measurement was taken with a Mexameter MX 18 probe. Analysis of TEWL was conducted in a continual way for 30 seconds. Skin pH was measured with a pH-Meter PH-90 probe. The mexametric probe assesses skin colour on the basis of melanin and haemoglobin contents.

### Statistical analysis

Statistical analysis was performed with the use of the non-parametric Kruskal–Wallis test by rank-sum for independent groups. For the values obtained, the median and quartile were calculated. The level of significance was P < 0.05. The calculations were made with the use of R for Windows software, version 3.5.5, produced by the R Foundation for Statistical Computing (Vienna, Austria) [14].

## Results

### Biophysical parameters of the skin in specific animal groups within the examined skin regions

#### Measurement of TEWL

Of the five examined skin regions, for the lips of stallions, the results were significantly higher than in geldings (P = 0.009) and mares (P = 0.023) (Table 1). In other examined regions, no differences were observed between the parameters assessed within the groups.

**Table 1:** The values of transepidermal water loss (TEWL) in the lip region.

Group	q1	median	q3
**Mare**^**a**^ **(n = 7)**	45.813	48.410	48.772
**Stallion**^**a**^^,^^**b**^ **(n = 7)**	63.130	64.947	66.727
**Gelding**^**b**^ **(n = 7)**	37.983	38.763	48.698

*Note*: a–statistically significant differences between mares and stallions, P = 0.023

b–statistically significant differences between stallions and geldings, P = 0.009.

#### Corneometry (CM)

**Fig 2:**
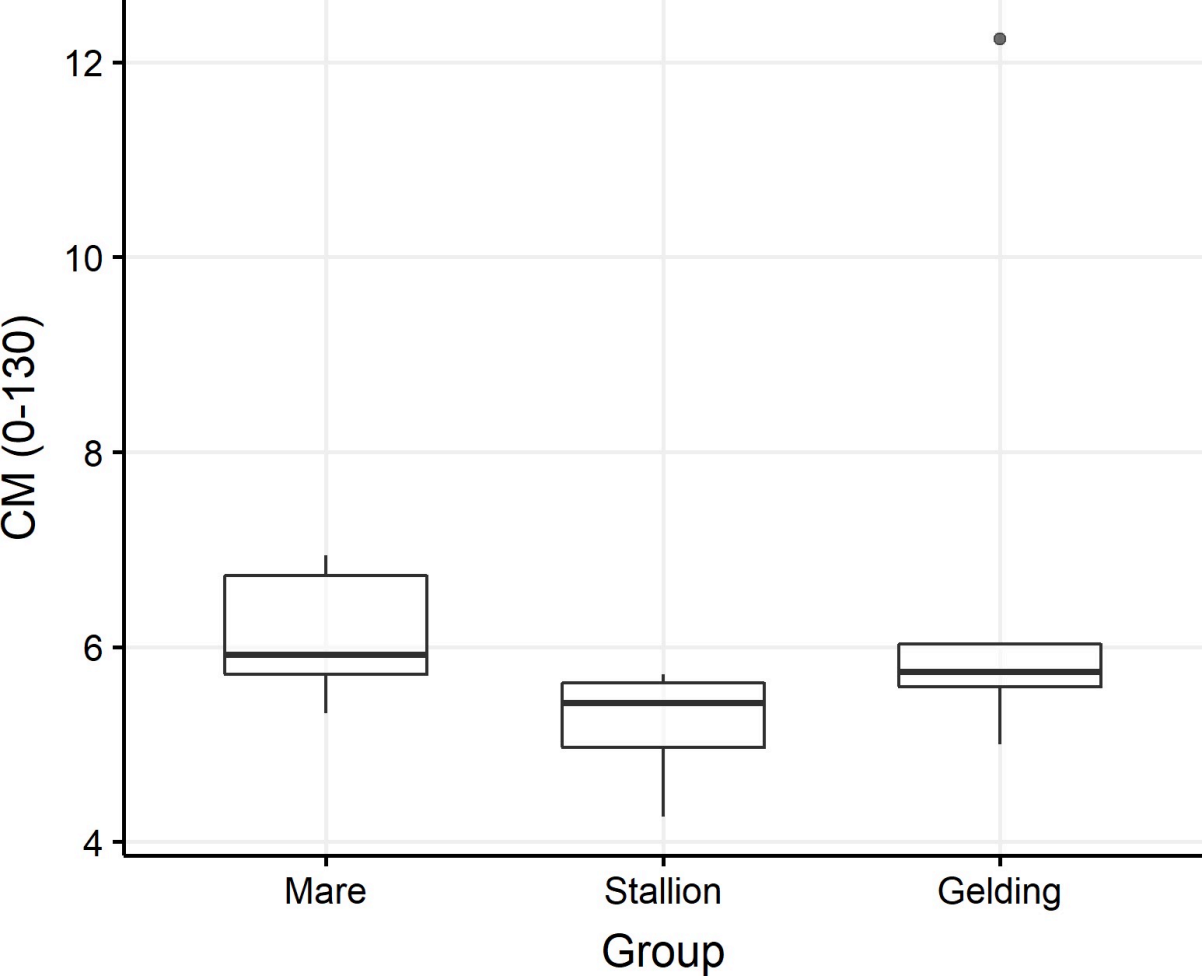
Corneometry values (CM) in the neck region. Data are expressed as a value in self units for the Corneometer^®^ CM 825 probe. P < 0.05. The results were significantly higher between mares and stallions.

**Fig 3:**
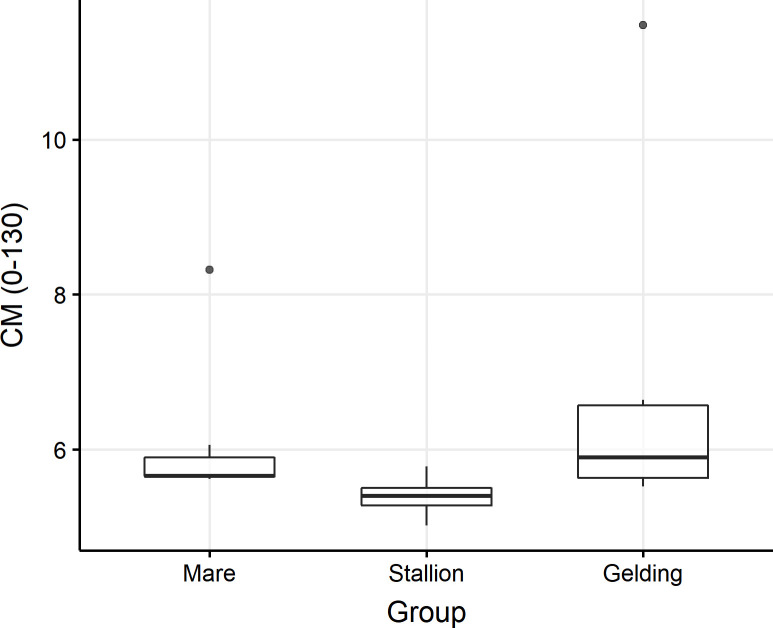
Corneometry values (CM) in the prosternum region. Data are expressed as a value in self units for the Corneometer^®^ CM 825 probe. P < 0.05. The results were significantly higher between mares and stallions and between stallions and geldings.

The results for mares, geldings and stallions in the neck region are presented in Fig 2, whilst the data concerning the prosternum are shown in Fig 3. Of the five studied regions, in the neck of mares, the results were significantly higher than in the stallions (P = 0.037). Additionally, in the prosternum, the results were significantly higher in mares compared with stallions (P = 0.037) and in geldings compared with stallions (P = 0.018). In the other locations, no statistically significant differences connected with the gender of the animals were seen.

#### Measurement of skin pH

Table 2 describes the results obtained in the region of the prosternum in mares, stallions and geldings. The results obtained in the five studied skin regions of the prosternum were significantly higher in stallions than in mares (P = 0.037). In the other areas, no statistically significant differences were observed in the groups studied.

**Table 2:** pH values in the prosternum region.

Group	N	q1	median	q3
**Mare**^**a**^	7	7.276	7.670	7.736
**Stallion**^**a**^	7	7.823	8.006	8.192
**Gelding**	7	7.509	7.666	7.889

*Note*: a–statistically significant difference between stallions and mares, P = 0.037.

#### Skin temperature measurement (thermometry)

Table 3 describes the results obtained in mares, stallions and geldings. Of the five regions studied, the results obtained in the prosternum in geldings were significantly higher than in stallions (P = 0.049). In the other areas, no statistically significant differences were observed in the groups studied.

**Table 3:** Temperature values (TH) in the prosternum region.

Group	n	q1	median	q3
**Mare**	7	30.940	31.24	31.89
**Stallion**^**a**^	7	30.143	30.68	30.92
**Gelding**^**a**^	7	31.160	31.34	31.86

*Note*: a–statistically significant differences between geldings and stallions: P = 0.042.

#### Mexametry (MX_MEXA and MX_ERYTH)

The results obtained did not reveal statistical differences between the groups or between the regions studied.

## Discussion

In this experiment, physicochemical parameters of the skin in mares, stallions and geldings were assessed in five skin regions. These skin regions were selected on the basis of published data, which allowed for more precise comparison of the results [6, 9, 10]. The examinations were performed on one horse breed, Polish Konik horses, so that the effect of breed on the assessment results could be eliminated. The hair was not shaven, as studies carried out so far have not indicated any effect of hair removal on the studied parameters [9]. In one study on unclipped hair, TEWL values were different and not that consistent as in clipped hair, but the result seems not to be ideal as it could be affected by the time of measurement [9]. In the presented study, the difference could be a result of time intervals, as the different values were obtained only four hours after the first measurement. Only clinically healthy individuals were examined, to eliminate the impact of skin diseases on the biophysical parameters [12, 13, 15, 16]. Additionally, it has been shown that ultrastructural skin lesions, occurring, among other things, secondary to atopic dermatitis might affect the physicochemical parameters of the skin in horses, and for this reason, all the horses within the study group did not demonstrate dermatological symptoms either during the examination or in the past [10].

Some earlier studies did not show any effect of horse gender on TEWL values [6, 17], which was partly confirmed by the present work. Some researchers suggest that hair length might have some effect on the TEWL results obtained, so the values of the parameters studied may vary between different breeds [17–19]. As the study group consisted of one horse breed, the hair length was not taken as a differentiating factor but it should be taken into consideration when comparing different body regions as there are no data regarding hair length in horses. Studies with measurements of hair length should be conducted as a next step. In the current study, a statistically significant difference in TEWL values was observed solely in the region of the lips–in stallions the values were higher than in geldings and mares. High TEWL values in the region of the lips may be logically explained given the thinner skin in this region than in other areas of the body. In the region of the lips and mandible, there are also more sweat glands [20–22]. Additionally, it is the region with increased moisture resulting from contact with the air coming from the nostrils and from the possibility of lip-licking by the animal. The lips are also covered with more delicate and shorter hair. This does not explain, however, why these values were increased only in the group of stallions. Studies carried out by Szczepanik et al. [4, 17] showed some lower TEWL values in the lumbar region than in other locations in horses. Additionally, some differences were observed both between breeds and body regions–in particular in the lumbar and inguinal regions [6]. However, this does not confirm the observations made by the authors of the present study, but can perhaps be explained by the selection of other horse breeds for those studies. TEWL can also be connected with temperature. This is still an issue that should be investigated, but a higher skin temperature can increase TEWL. Skin temperature is significantly higher in the head area (eyes, lips) and neck area. Studies conducted by Szczepanik revealed that not only body region influences TEWL values but also breed [6]. In that case, comparing results from different horse breeds can be misleading and further investigation is required.

CM parameters can be affected not only by the thickness of the epidermis, but also the structure of the skin components, the number of sweat and sebaceous glands, as well as ultrastructural skin lesions occurring secondary to various diseases. CM values also change in the case of skin damage (including burns), diseases occurring with epidermal exfoliation and atopic dermatitis (AD) [12], which may be applied, among other things, in the evaluation of the progress of local and systemic therapies. Studies performed in humans have not shown any effect of hair shaving on survey results [23]. Studies in dogs rendered contradictory results concerning the impact of the breed on corneometry results, although this may have been a result of the examination technique and the possibility of dogs’ moving, their living conditions and diet [3, 24]. A literature overview suggests that the present study is the first to describe the measurement of corneum hydration in horses. The examinations carried out by the authors show that the results for the side of the neck were significantly higher in mares than in stallions. Additionally, in the region of the prosternum, the results were significantly higher in mares and geldings when compared with stallions. Given the lack of published data, it is difficult to interpret the results obtained as a specific indication of a relationship between gender and skin region, a fact which encourages continued studies in this respect.

Skin pH varies depending on animal species and the course of skin diseases, as it is one of the integral constituents of the epidermal barrier [25]. It has been observed that skin pH increases in pyoderma in dogs [5]. Moreover, there have been studies that showed an effect of diet on pH in the case of feline skin [26]. Skin pH may be a specific biomarker in inflammatory skin conditions, including pyoderma or *Malassezia* dermatitis–in such cases, the proliferation of yeast-like fungi causes an increase in pH. Conversely, a decrease in skin pH is useful in the treatment of skin yeast infections and infections of the external ear canal [2]. In horses, the skin has an alkaline reaction ranging between 7.2–7.5, so excessive acidification may lead to skin irritation and damage to the epidermal barrier. In this animal species, problems with candidiasis are not frequently reported, but bacterial skin inflammation occurs quite often, especially secondary to traumas or skin macerations resulting from poor environmental conditions. Studies of skin pH in horses carried out so far have revealed differences in the values of this parameter in various skin regions–differences between the neck and the scapular region or the lip region, between the lip region and the neck region, the shoulder region, the thoracic region, the lumbar region and the inguinal region [17]. In horses, no effect of gender on skin pH has been demonstrated so far [17]. In the present study a statistically significant difference was found only in the region of the side of the chest–in mares the values were higher than in stallions, which only partly corresponds to results obtained by other specialists. In studies performed with a group of German shepherd dogs, similar results were rendered in all examined regions of the body in males and females, but generally, these results were higher than in other breeds, which might prove the effect of breed on the parameter studied [27]. This points to the possibility of breed-related differences but does not confirm the hypothesis that males and females differ significantly, and this fact was also confirmed by other authors.

The mexametry results obtained (MX_MEXA and MX_ERYTH) did not reveal any statistically significant differences between the groups or the studied regions. This result is indicative of the fact that, in each group and each examined region, there were no differences in the melanin and haemoglobin content in the epidermis. Perhaps this examination may also be applied in horses with vitiligo. It is necessary, however, to establish the melanin content in healthy skin for any given breed (skin type).

The data obtained in probe thermometry were compared between stallions, mares and geldings. Of the five examined skin regions, in the prosternum, the results were significantly higher in geldings than in stallions. To the best of our knowledge, such an extensive skin assessment with the use of a thermometric probe has not been carried out previously. The available data and comparisons of temperature in various skin regions were obtained solely with the use of classical thermometry. Changes in blood circulation are responsible for temperature variations for a given skin region and the surrounding soft tissue. There are published data available concerning skin temperature in horses and other animal species, yet all these assessments were made with the use of thermography [28, 29]. These studies suggest that this is a good indicator in the case of evaluation of the treatment of inflammation and the progress of rehabilitation in sporting horses as well as in the assessment of traumas in other animal species [29]. Moreover, it is possible to apply this examination for the prediction of mastitis and treatment evaluation in lactating mares. Studies that have been performed comparing the values on the pelvic limbs in healthy dogs proved the accuracy and comparability of the method [30]. It is known that in the case of dermatitis, skin temperature is locally elevated, a fact which is certainly the outcome of ongoing skin inflammation and this is why thermometry may also be applied for dermatological assessments. This method may be used in the case of various types of skin inflammation–infectious (phlegmon) or allergenic. Thermography certainly differs from thermometry, which was applied here, but in the current experiment, selected body regions were examined, allowing more accurate results to be obtained. It must be observed that the reports published so far point to a lack of correlation between surface and rectal temperature [31]. The fact that corneometry and thermometry in geldings rendered significantly higher results with respect to the prosternum than other body areas in comparison with stallions, is not completely clear for the authors and requires further, more detailed research.

The research, as carried out by the authors, definitely has the character of a pilot study concerning animal breed and the extensive scope of physicochemical skin assessment. To the authors’ knowledge, so far, such a comprehensive and thorough study describing biophysical skin properties in horses, including Polish Konik horses, has not been published, a fact which encourages further studies in this respect. In this situation, one serious drawback is the difficulty in verification of the results and comparison with other studies on horses, with respect to the limited data. Yet, the example of human dermatology, where research is still being carried out, gives some hope for the development of the field being studied, and for its future application in veterinary medicine.

## Supporting information

S1 Fig The values of the transepidermal water loss (TEWL) in the lip region. Data are expresses as a value of transepidermal water loss in gram per hour per square meter (g/h/m2). P < 0,05. The results were significantly higher in lip area of stallions in compare to geldings and mares.
(TIF)

S2 Fig Coronometry values (CM) in the neck region. Data are expresses as a value in self units for Corneometer® CM 825 probe. P<0,05. The results were significantly higher between mare and stallion.
(TIF)

S3 Fig Coronometry values (CM) in the prosternum region. Data are expresses as a value in self units for Corneometer® CM 825 probe. P<0,05. The results were significantly higher between mare and stallion and between stallion and gelding.
(TIF)

S4 Fig The pH values in the prosternum region. Data are expressed in pH scale. P < 0,05. The results showed statistically significant differences between stallion and mare.
(TIF)

S5 Fig Temperature values (TH) in the prosternum region. Data are expressed in Celsius degrees. P<0,05. The results showed statistically significant differences between geldings and stallions.
(TIF)
