# Supplementary figures and images for: The examination of biophysical parameters of the skin in Polish Konik horses
Source: PLoS One. 2021 Jun 21;16(6):e0250329. doi: 10.1371/journal.pone.0250329 (PMC8216546; doi:10.1371/journal.pone.0250329)

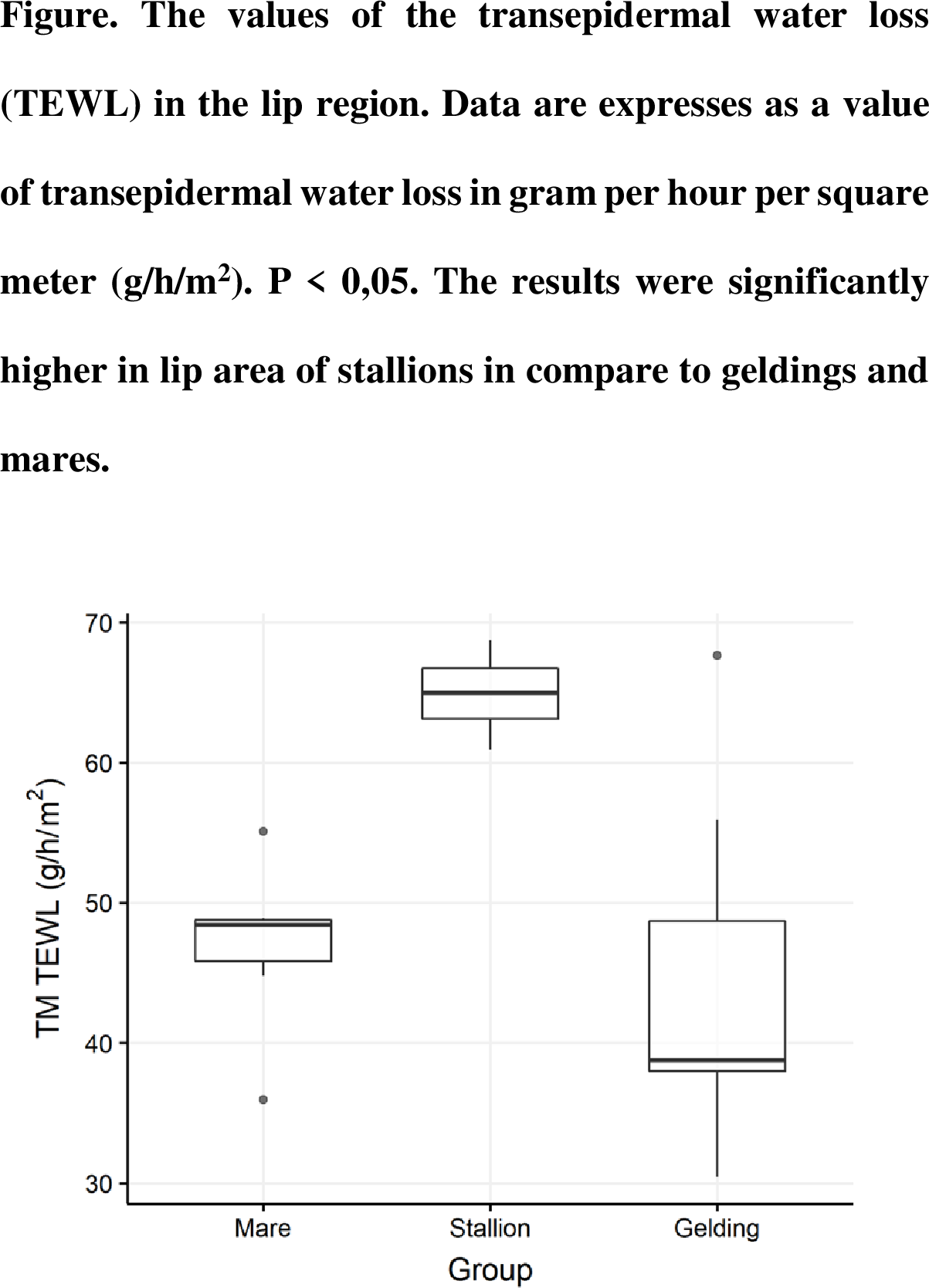

Supplement: S1 Fig — Data are expresses as a value of transepidermal water loss in gram per hour per square meter (g/h/m2). P < 0,05. The results were significantly higher in lip area of stallions in compare to geldings and mares. (TIF) [file pone.0250329.s001.tif]

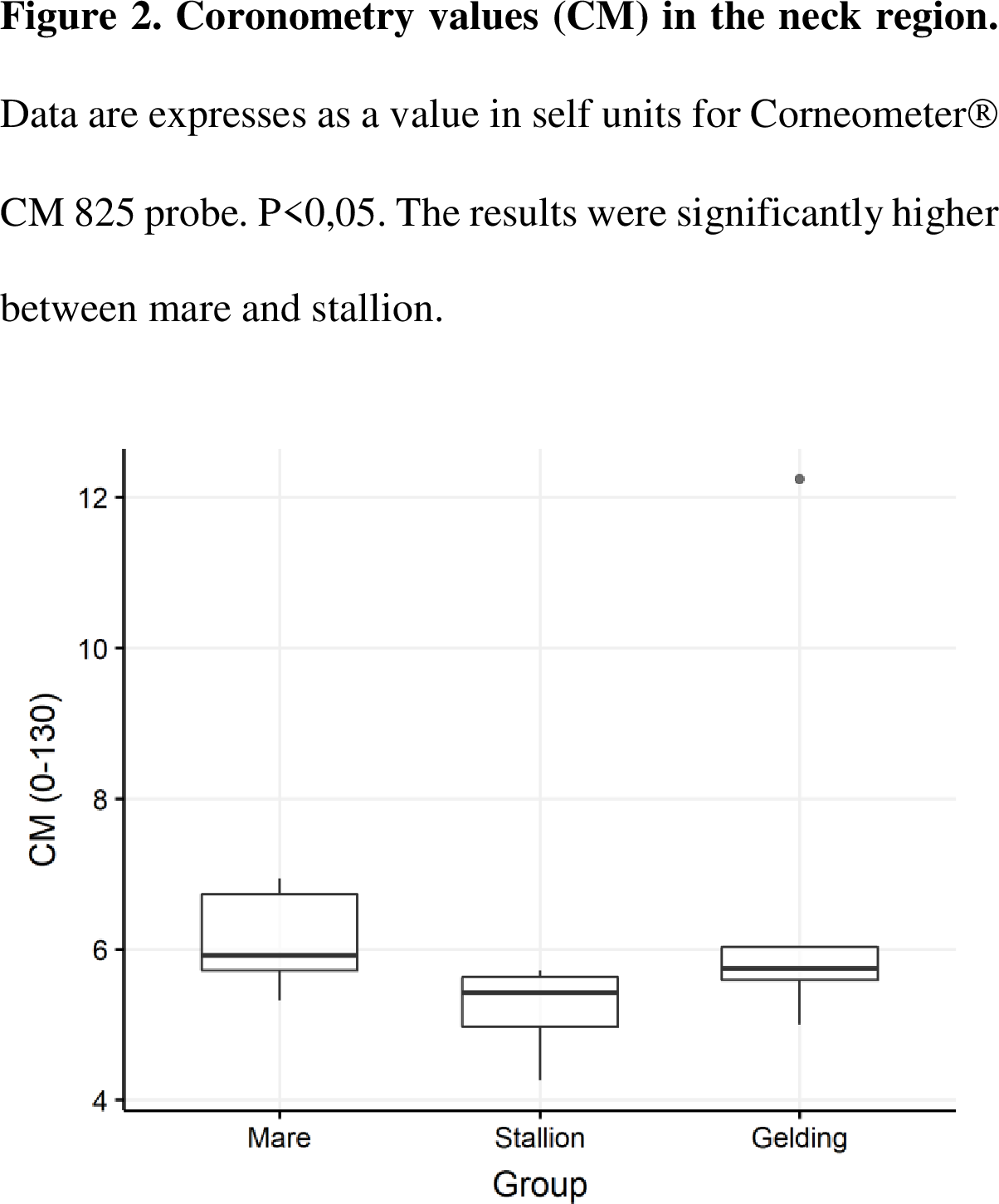

Supplement: S2 Fig — Data are expresses as a value in self units for Corneometer® CM 825 probe. P<0,05. The results were significantly higher between mare and stallion. (TIF) [file pone.0250329.s002.tif]

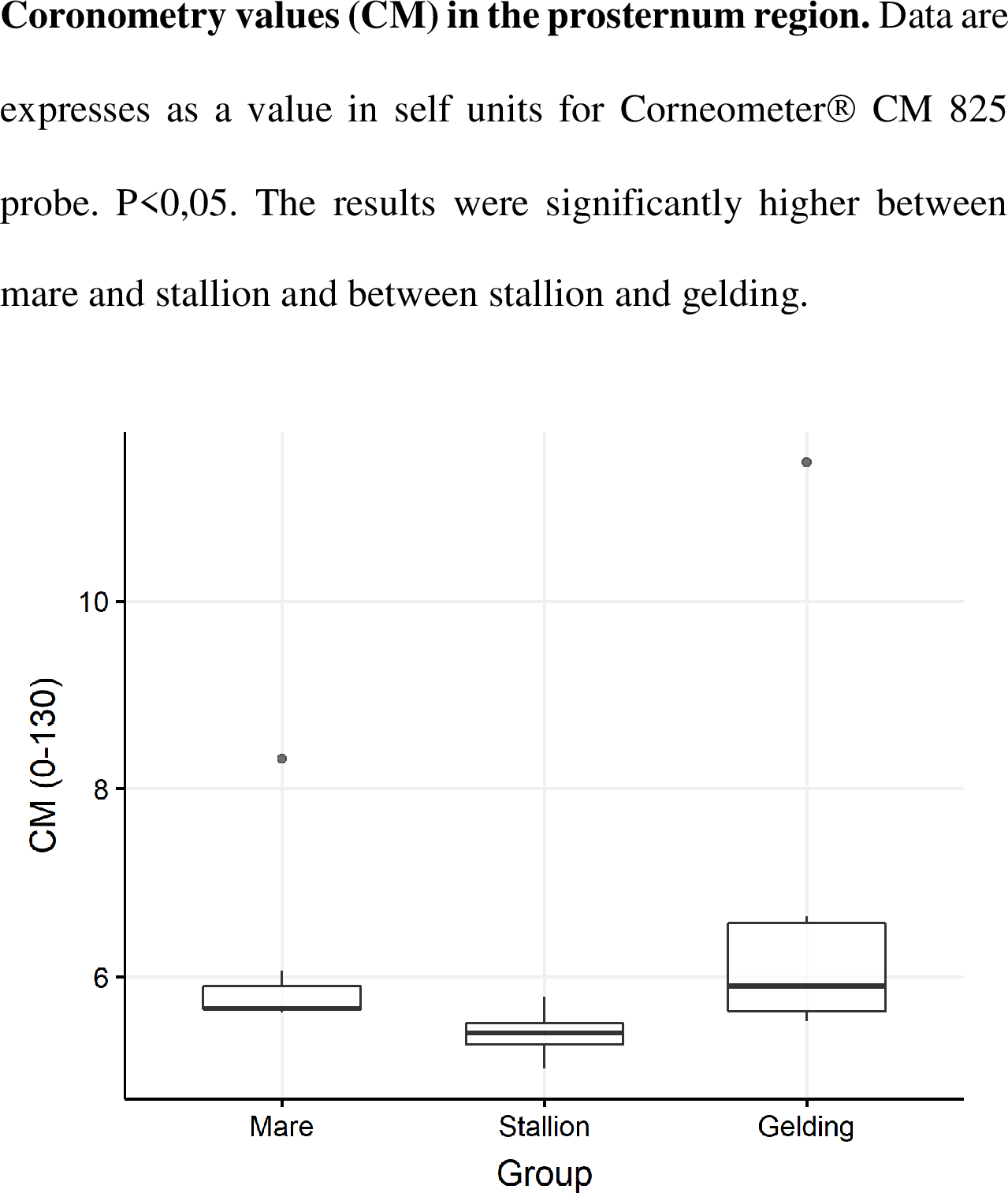

Supplement: S3 Fig — Data are expresses as a value in self units for Corneometer® CM 825 probe. P<0,05. The results were significantly higher between mare and stallion and between stallion and gelding. (TIF) [file pone.0250329.s003.tif]

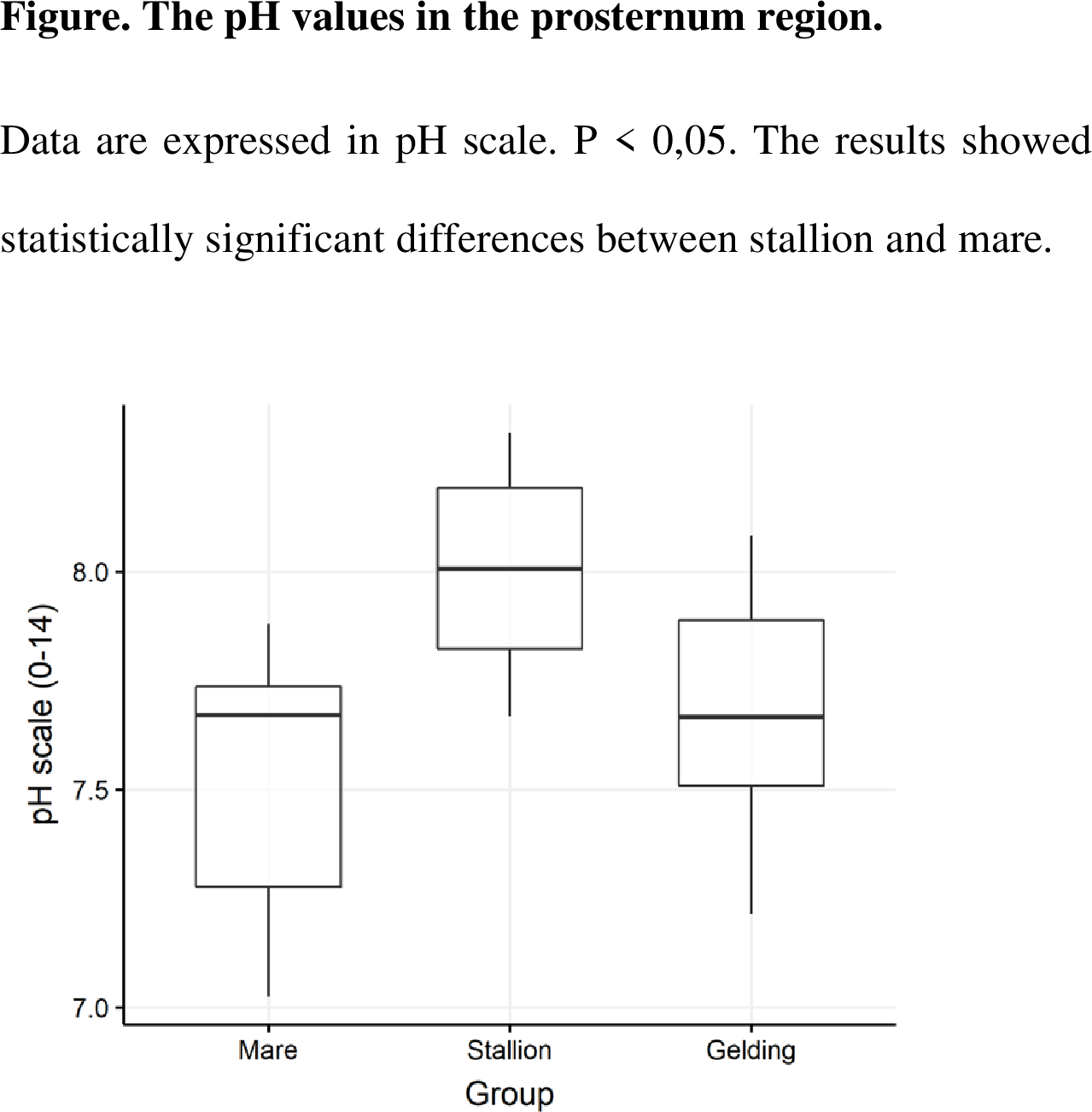

Supplement: S4 Fig — Data are expressed in pH scale. P < 0,05. The results showed statistically significant differences between stallion and mare. (TIF) [file pone.0250329.s004.tif]

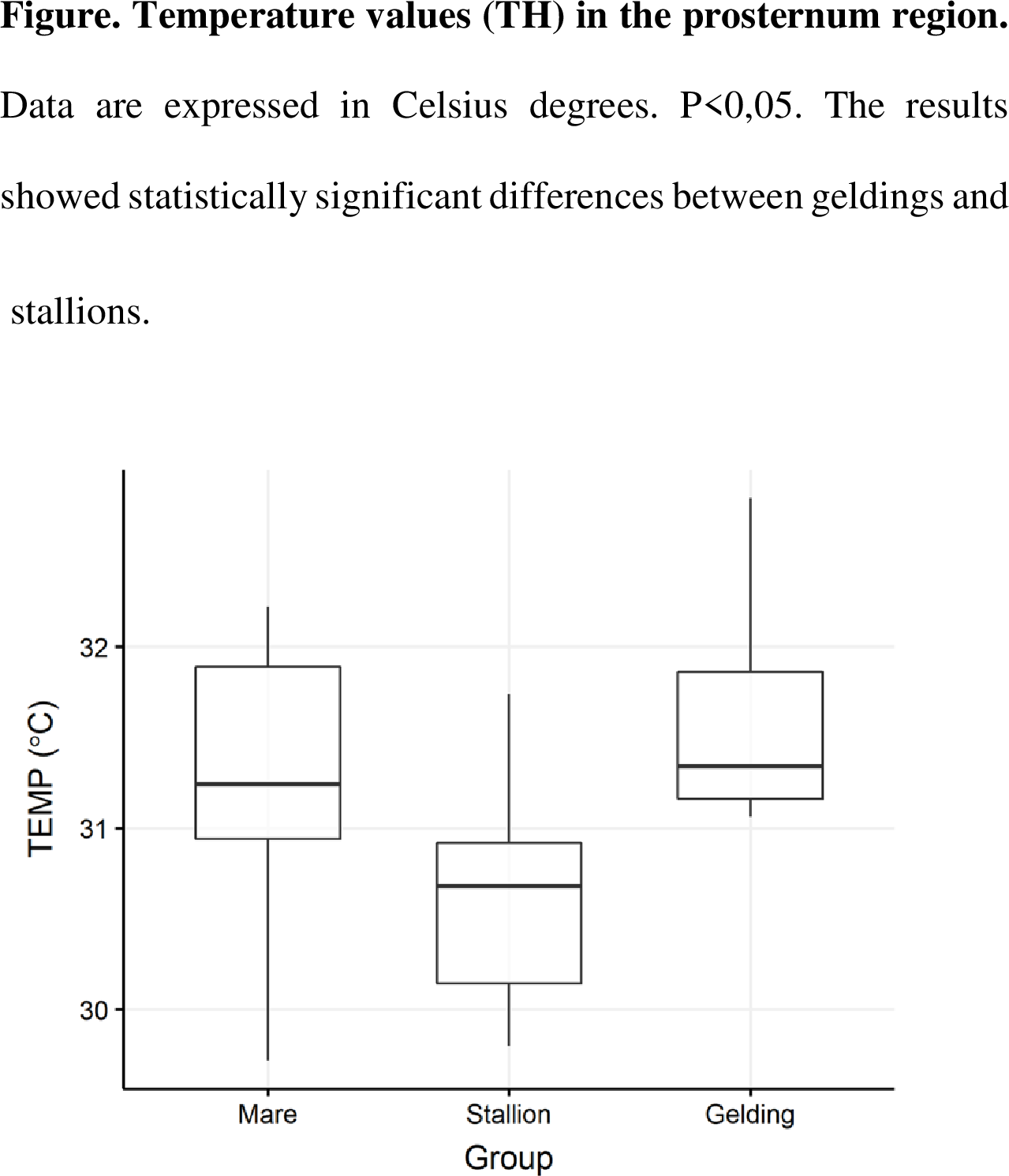

Supplement: S5 Fig — Data are expressed in Celsius degrees. P<0,05. The results showed statistically significant differences between geldings and stallions. (TIF) [file pone.0250329.s005.tif]
